# Tear Film Break-Up Time and Dry Eye Disease Severity in a Large Norwegian Cohort

**DOI:** 10.3390/jcm10040884

**Published:** 2021-02-22

**Authors:** Mazyar Yazdani, Jørgen Fiskådal, Xiangjun Chen, Øygunn A. Utheim, Sten Ræder, Valeria Vitelli, Tor P. Utheim

**Affiliations:** 1 The Norwegian Dry Eye Clinic, 0366 Oslo, Norway; jorgen_fiskaadal@hotmail.com (J.F.); chenxiangjun1101@gmail.com (X.C.); outheim@gmail.com (Ø.A.U.); raeder.sten@gmail.com (S.R.); utheim2@gmail.com (T.P.U.); 2 Department of Medical Biochemistry, Oslo University Hospital, 0450 Oslo, Norway; 3 Department of Oral Surgery and Oral Medicine, University of Oslo, 0317 Oslo, Norway; 4 Department of Ophthalmology, Sørlandet Hospital Arendal, 4604 Arendal, Norway; 5 Department of Ophthalmology, Oslo University Hospital, 0450 Oslo, Norway; 6 Oslo Center for Biostatistics and Epidemiology, Department of Biostatistics, University of Oslo, Sognsvannsveien 9, 0372 Oslo, Norway; valeria.vitelli@medisin.uio.no; 7 Department of Ophthalmology, Stavanger University Hospital, 4011 Stavanger, Norway; 8 Department of Plastic and Reconstructive Surgery, Oslo University Hospital, 0450 Oslo, Norway

**Keywords:** dry eye disease, tear film break-up time (TFBUT), cut-off values

## Abstract

This study evaluated to what extent tear film break-up time (TFBUT) could discriminate pathological scores for other clinical tests and explore the associations between them. Dry eye patients (*n* = 2094) were examined for ocular surface disease index (OSDI), tear film osmolarity (Osm), TFBUT, blink interval, ocular protection index (OPI), ocular surface staining (OSS), Schirmer I test, meibomian expressibility, meibomian quality, and meibomian gland dysfunction. The results were grouped into eight levels of break-up time (≤2, ≥3, ≤5, ≥6, ≤10, ≥11, ≤15, and ≥16) with or without sex stratification. Receiver-operating characteristic curve (ROC) analysis and Pearson’s correlation coefficients were used to study TFBUT’s discriminative power and the associations among the tests, respectively. Above and below each TFBUT’s cut-off, all of the parameters indicated significant difference between groups, except OSDI (cut-off 15 s) and Osm (cut-offs 5 s–15 s). At TFBUT cut-off of 2 s, sex difference could be detected for OSDI, Osm, and OSS. OPI presented the strongest discriminative power and association with TFBUT in sharp contrast to Osm, holding the poorest discriminative power with no significant correlation. The remaining parameters were within the poor to very poor categories, both with regard to discrimination and correlation. In conclusion, patients with lower TFBUT presented with more severe DED parameters at all four defined cut-off values.

## 1. Introduction

Dry eye disease (DED) with prevalence of 5–50% of the population is one of the most common diseases, not only in ophthalmology, but in medicine in general. Symptoms may include irritation, dryness, foreign body sensation, pain, photophobia and blurred vision [1,2]. Although a variety of questionnaires and clinical tests have been developed, there is no general agreement on the most efficient method for diagnosis of DED. For example, a combination of Schirmer I test (ST) and tear film break-up time (TFBUT) [3], joint results of OSDI, ST and TFBUT [4], or concomitant outcomes of several clinical tests and questionnaires [5,6] have been previously suggested to increase the diagnostic predictability. In most papers, TFBUT plays a central role and, hence, is worth further investigation [7].

Assessment of tear film stability provides valuable information to clinicians, as a stable preocular tear film is a hallmark of ocular health. Since introducing fluorescein-based TFBUT by Norn [8] in 1969, a variety of techniques, such as non-invasive break-time, topography, interferometry, aberrometry, and visual function tests have been used to determine tear film instability. Nevertheless, TFBUT still remains the most frequently used and standard clinical test for estimating tear film stability [9,10,11].

For invasive TFBUT, the sodium fluorescein is installed into the tear film using a moistened strip or a pipette and its integrity is examined by a biomicroscope that is equipped with cobalt blue light and a wratten 12 yellow barrier filter [12,13]. In spite of its popularity, a major drawback of TFBUT is poor reproducibility and accuracy [14,15]. Additionally, the analyses might be influenced by the order of tests performed, the skill level of practitioners, cooperation of patients (partial vs. complete blinking), properties (preservatives, pH and concentration), and, importantly, the volume of fluorescein applied. Nonetheless, TFBUT is considered to be a valid ophthalmological test for the diagnosis of DED [9,16].

The association between TFBUT and blink interval (BI) ensures ocular surface protection. The break-up evolves in a characteristic way with time, as the longer the interval between break-up of the tear film and the subsequent blink, the greater susceptibility to ocular surface damage [17]. Despite diurnal and inter-individual variations [16], TFBUT is approximately ranged 3 –132 s (mean: 30 s) [8]. For the diagnosis of DED, cut-offs of ≤10 s [13,18], 5–10 s, and ≤5 s [19,20,21,22] have been previously reported. Therefore, it is interesting to study how the choice of different reference values would affect the diagnostic outcomes.

The parameters collected from a large Norwegian cohort were in the present study grouped into eight levels of break-up time (using ≤2 s, ≤5 s, ≤10 s, and ≤15 s as cut-off values) with or without sex stratification in order to evaluate whether the TFBUT can be employed as a predictor for other dry eye tests. Additionally, receiver-operating characteristic curve (ROC) analysis was used to assess to what extent the TFBUT can be utilized as a discriminative test to obtain pathological scores for other tests. Finally, the associations among them were examined further while using correlation coefficients.

## 2. Materials and Methods

### Patients

A total of 2094 DED patients with different etiologies were consecutively recruited at the Norwegian Dry Eye Clinic between 2013 and 2018. Of the recorded data, 2019 subjects had measurements of both age and sex, 42 of sex only, three of age only, and 30 of neither age nor sex. Analysis was only performed for those with recorded TFBUT (1959 subjects), unless it is limited by the number of records for other clinical tests: Osm (*n* = 835), ST (*n* = 1942), meibomian expressibility (ME) (*n* = 1873), meibomian quality (MQ) (*n* = 1871), and meibomian gland dysfunction (MGD) (*n* = 1881) (Table 1). During the course of recruitment in The Norwegian Dry Eye Clinic, since 2013 extensive data have been collected and transformed into data sets to address specific research questions in various publications e.g., [5,23].

The Regional Committee for Medical & Health Research Ethics, Section C, South East Norway (REC) reviewed the use of the data for this study. REC found the research project “Evaluation of data from the Norwegian Dry Eye Clinic” to be outside the scope of the Act on Medical and Health Research (2008) and therefore could be implemented without its approval (REC ref: 2013/812, IRB ref: IRB00001870, Date: 4 June 2015). Informed consent was received from all patients prior to the collection of data. All the data collected from questionnaires and clinical tests were anonymized. All of the procedures performed in this study were in compliance with the Declaration of Helsinki.

## 3. Clinical Evaluation

Before any clinical tests, the patient filled out the OSDI questionnaire (Allergan Inc., Irvine, CA, USA) [24]. The tests were performed during regular working hours (09:00 to 16:00) and then systematically performed in the following order: (1) Osm measurement using a TearLab Osmolarity System (TearLab Corp, San Diego, CA, USA); (2) TFBUT through instillation of 5 µL 2% fluorescein sodium followed by a slit-lamp examination, in which the patient blinks three times and the average time from the last blink until first tear film break-up is recorded; (3) ocular protection index (OPI) by calculating the ratio of TFBUT divided by BI [25], an OPI value of less than 1 implies that tear film break-up occurs within the BI; (4) ocular surface staining (OSS) using fluorescein, utilizing the Oxford Grading Scheme (0–15; corneal staining 0–5) [26]; (5) ST without anaesthesia by installing paper strips on lower eyelid margin for 5 min.; (6) ME evaluation using slit lamp by measuring number of glands excreting meibum in the lower lid with light pressure from a cotton tip (0 = all five glands expressing meibum, 1 = 3–4 glands expressing, 2 = 1–2 glands expressing, and 3 = no expression from any gland); and, (7) MQ assessment by inspecting and scoring the quality of meibum secreted from the central eight glands of the lower lid from 0 to 3 using slit lamp (0 = clear, 1 = cloudy, 2 = cloudy with debris, 3 = thick, toothpaste-like). The sum of these glands is then added up to make out the final score (0–24). Finally, MGD were diagnosed according to the suggestions by the International workshop on MGD [27].

## 4. Statistical Analysis

Data from the right eye were used for statistical analysis. Descriptive statistics were performed in order to obtain test characteristics and subject demographics. For intergroup comparison of clinical parameter results based on cut-off values of TFBUT, the cohort was divided into below and above 2, 5, 10, and 15 s (eight groups). In addition, each group was further stratified according to sex. The Mann–Whitney test and Chi-Square test were used for inter- and intra-group comparison. The ability of the TFBUT to discriminate pathological score for each clinical test was analyzed by ROC analysis. This study considered optimum balanced sensitivity and specificity being values close to 50%. Pearson’s correlation coefficients of determination were calculated (*r*) between each test. All of the statistical analyses were executed using the R software (version 4.0.2) [28]. Data are presented as mean and standard deviation. The values of *p* < 0.05 were considered to be statistically significant.

## 5. Results

The overall average age of the patients was 52.7 ± 16.5 years (range: 2–95). For females, average age was 54.2 ± 15.7 (range: 13–94) and for males it was 48.6 ± 17.9 (range: 2–95). The Mann–Whitney test for differences in the age distribution between sexes gave a *p*-value < 0.001. However, the distribution across sex was similar.

Table 1 provides the overview of sample size (also stratified with respect to sex) for each test. Except Osm (*n* = 835), the rest contains 1759–1977 patients. The number of females surpassed the males in all test groups. The test data distribution is significantly (<0.05) different between males and females for OSDI, Osm, TFBUT, BI, OPI, and OSS, but not others. When subjects were categorized into age groups (Table 2), the number of subjects from lowest to highest were 0–19 (*n* = 28), 80–99 (*n* = 78), 20–39 (*n* = 451), 60–79 (*n* = 679), and 40–59 (*n* = 783). In all but one group (0–19), female patients outnumbered male patients. The proportion of subjects in each age group was significantly different between sexes (< 0.05) for all but one group (80–99).

Table 3 shows distribution of TFBUT above and below each cut-off value. Among the participants, 42.58% (1.56 ± 0.5), 77.62% (2.56 ± 1.2), 94.06% (3.44 ± 2.3), and 97.88% (3.81 ± 2.9) had a TFBUT test of <2, ≤5, ≤10 and ≤15 s, respectively. The parameters categorized based on four TFBUT cut-off values (Table 4) indicated significant difference (<0.05) between groups above and below each cut-off value. However, the only exceptions belonged to OSDI (cut-off 15 s) and Osm (cut-offs 5 s, 10 s, and 15 s). Further, sex stratification (Table 5) revealed non-statistical significance (≥0.05) for OSDI (≥6 s, ≥11 s and ≥16 s), Osm (≥6 s, ≥11 s and ≥16 s), BI (≤2 s, ≥6 s, ≥11 s and ≥16 s), OPI (all except ≥3 s), ST and OSS (≥6 s, ≥11 s and ≥16 s), ME (all except ≥6 s), and MQ and MGD.

**Figure 1:**
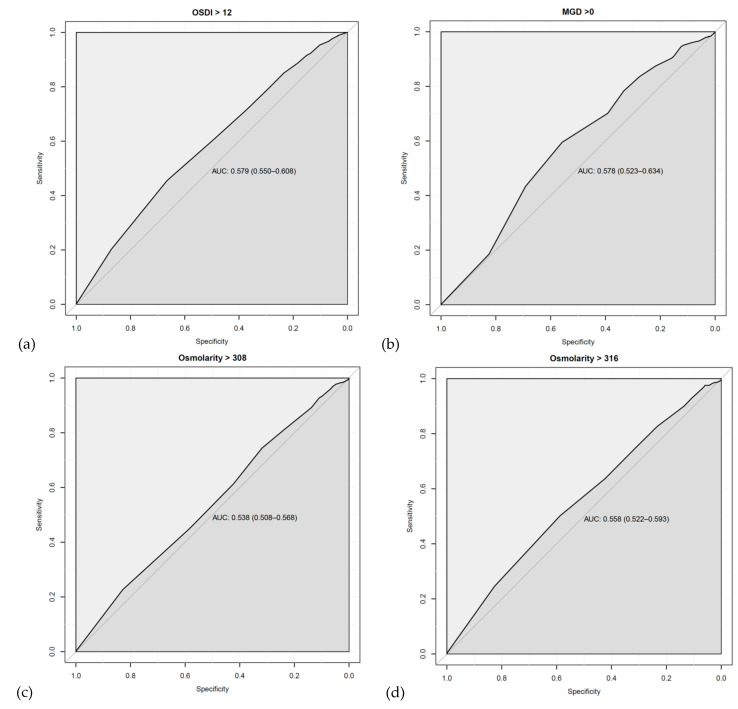

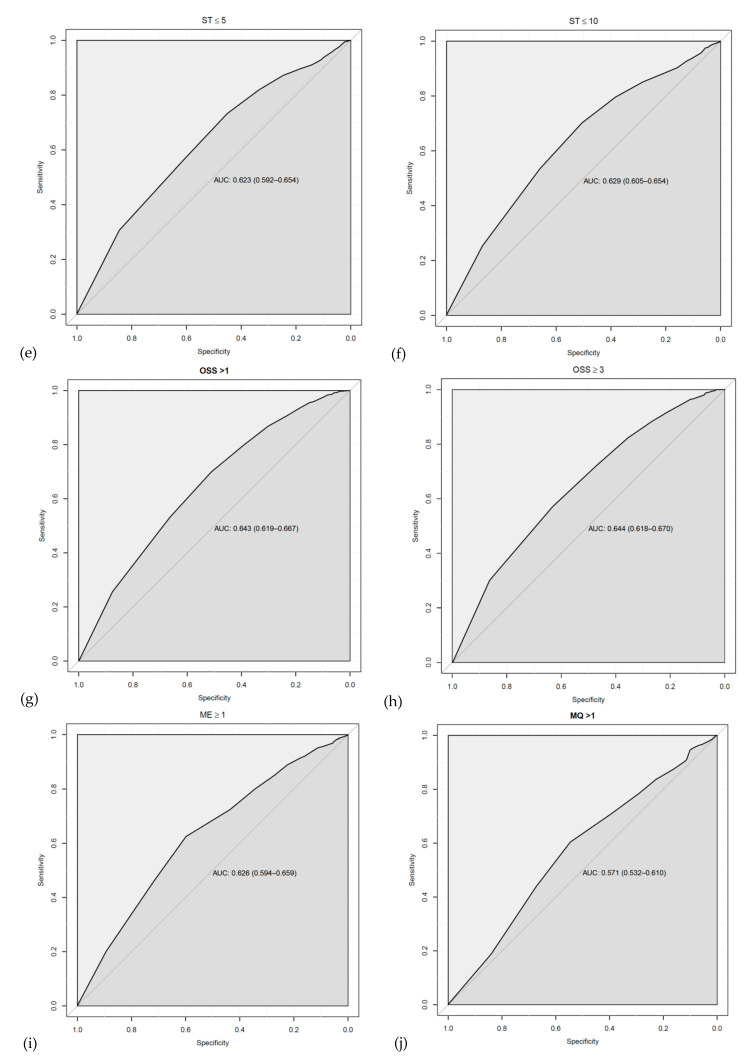

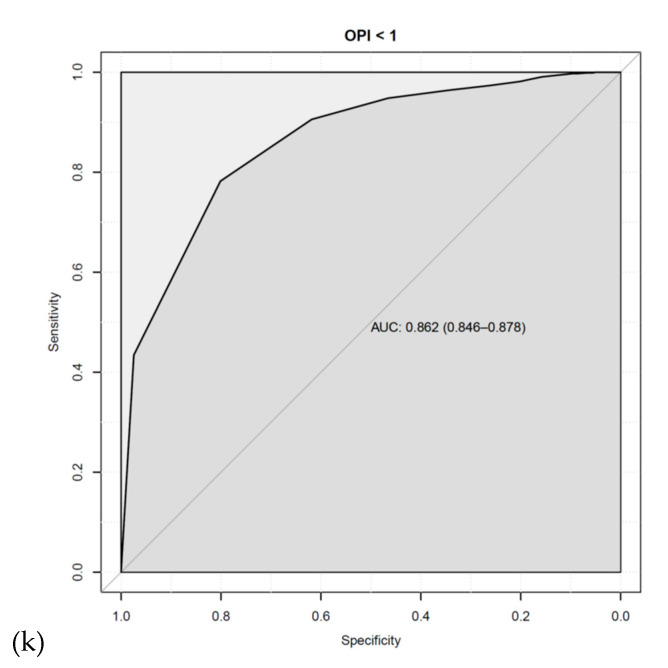
Receiver-operating characteristic curve (ROC) curves (with AUC and associated 95% confidence interval) are displayed to determine the optimum balanced sensitivity and specificity of TFBUT to predict pathological ocular surface disease index (OSDI) >12 (AUC = 0.579, 0.550 – 0.608) in subfigure (**a**); meibomian gland dysfunction (MGD) >0 (AUC = 0.578, 0.523 – 0.634) in subfigure (**b**); osmolarity >308 (AUC = 0.538, 0.508 – 0.568) in subfigure (**c**) and >316 (AUC = 0.558, 0.522 – 0.593) in subfigure (**d**); Schirmer I test (ST) ≤5 (AUC = 0.623, 0.592 – 0.654) in subfigure (**e**) and ≤10 (AUC = 0.629, 0.605 – 0.654) in subfigure (**f**), ocular surface staining (OSS) >1 (AUC = 0.643, 0.619 – 0.667) in subfigure (**g**) and ≥3 (AUC = 0.644, 0.618 – 0.670) in subfigure (**h**), meibum expressibility (ME) ≥ 1 (AUC = 0.626, 0.594 – 0.659) in subfigure (**i**); meibum quality (MQ) >1 (AUC = 0.571, 0.532 – 0.610) in subfigure (**j**); and ocular protection index (OPI) <1 (AUC = 0.862, 0.846 – 0.878) in subfigure (**k**).

Figure 1 presents the results of ROC curve analysis. The highest calculated AUC (86.2%; *p* < 0.0001) was obtained by TFBUT cut-off value of 2.5 s for OPI < 1 (sensitivity 78.2% and specificity 80.1%). The second highest AUC was calculated for OSS ≥ 2 (64.4%; *p* < 0.0001) by TFBUT cut-off value of 2.5 s, followed by OSS > 1 (64.3%; *p* < 0.0001), ST ≤ 10 (62.9%; *p* < 0.0001), ME ≥ 1 (62.6%; *p* < 0.0001), and ST ≤ 5 (62.3%; *p* < 0.0001) by TFBUT cut-off time of 3.5 s.

TFBUT was significantly correlated to all variables (<0.0001), excluding Osm (*p* = 0.0527), as shown in Table 6. It was positively correlated to OPI (*r* = 0.696), ST (*r* = 0.205) and BI (*r* = 0.093). In contrast, the negative correlations were observed between TFBUT and OSS (*r* = −0.241), ME (*r* = −0.191), MGD (*r* = −0.134), MQ (*r* = −0.108), and OSDI (*r* = −0.091). Among other tests (Table 7), no *r* above 0.2 was recorded, except for meibomian gland tests. MGD was positively correlated to ME (*r* = 0.289) and MQ (*r* = 0.259).

## 6. Discussion

DED includes a broad range of alterations in the quality or quantity of tear film with different etiology and pathophysiology. Symptoms, ocular surface abnormalities, and tear abnormalities are three main proposed criteria for the diagnosis of DED [29]. For the latter, tear film stability using TFBUT is one of the most common examinations. Like other clinical tests, TFBUT suffers from the lack of well-defined cut-off values, leading to mixed recommendations on the guidelines for DED diagnosis [30,31]. This study, with a large Norwegian cohort of DED patients, showed that patients with lower TFBUT presented with more severe DED at all four defined cut-off values. Using cut-off of ≤2 s, the value that was below the range of variations reported for TFBUT (3–132 s, mean: 30 s) [8], almost half of recruited subjects (42.58%) fell into pathological category (Table 3). Although a close value (<3 s) has been previously reported for screening [32], such a criterion seems to be useful in extreme cases of DED. For cut-offs of ≤5 s and ≤10 s, a 20% difference in detection clearly highlights the possibility of overlooked or missed subtle cases. The application of larger cut-off value, ≤15 s, included a majority of our recruited patients (97.88%). With the recommended values by the Japan Dry Eye Society and the Asia Dry Eye Society (≤5 s) [33], 1502 of our patients were included. Using the Dry Eye Workshop II recommendation (≤10 s) [34], the number of screened individuals increased to 1820. Therefore, considering 5–10 s as the marginal cut-off range [21] seems to be appropriate.

All of the parameters, except OSDI (cut-off 15 s) and Osm (≤5 s to ≤15 s categories), which were grouped based on the four cut-offs of TFBUT could significantly discriminate pathological DED (Table 4). Using ROC analysis, although the discriminative ability of TFBUT for OSDI > 12 (57.9%; *p* < 0.0001), MGD > 0 (57.8%; *p* = 0.00177), MQ > 1 (57.1%; *p* = 0.0002), Osm > 316 (55.8%; *p* = 0.0008), and Osm > 308 (53.8%; *p* = 0.0066) was significant, the performance was very poor (AUC 0.5–0.6) [35]. OPI presented the strongest discriminative power as well as association with TFBUT in the present study. It was expected, as TBUT is one of two factors in the OPI equation. The interaction between TFBUT and BI is critical to the health of the ocular surface. Therefore, OPI was developed to quantify relationship between these parameters through dividing TFBUT by IBI [25]. Hence, the application of OPI has been reported for several observational studies and clinical trials [5,36,37,38,39,40,41].

Osm grouped based on TFBUT cut-offs of ≤5 s, ≤10 s, and ≤15 s was unable to significantly discriminate the pathological score. Further examination using ROC analysis also indicated the poorest discriminative power for Osm when compared to other tests. These were in line with the lack of significant correlation between TFBUT and Osm (Table 6). The absence of such an association has been previously reported in primary Sjögren’s syndrome [42] and DED patients [43]. Interestingly, Yeh et al. [44] could not find any clinical association between them, although their statistical analysis showed that higher tear osmolarity was significantly associated with longer TFBUT. Several studies have questioned the diagnostic ability of osmolarity [45,46,47,48,49], but others have found osmolarity to have high diagnostic capacity [50,51]. Thus, more research on the role of osmolarity in diagnostics, including cut-off values and other biochemical correlations, are warranted.

Other variables have been either positively (ST and BI) or negatively (OSS, meibomian gland function parameters and OSDI) correlated to TFBUT. No *r* > 0.2 was observed, except OSS (*r* = −0.241) and ST (*r* = 0.205). In a comparable study, Sullivan et al. [52] reported correlation coefficients between TFBUT and other tests: Osm (*r^2^* = 0.06), ST (*r^2^* = 0.08), fluorescein corneal staining (*r^2^* = 0.14), lissamine green conjunctival staining (*r^2^* = 0.15), Bron/Foulks meibomian gland grading (*r^2^* = 0.15), and OSDI (*r^2^* = 0.09) in 344 subjects with (*n* = 262) and without (*n* = 82) DED. For comparison, the authors used an independent data set with 200 subjects (184 DED; 16 controls) [45]. Their analysis showed a correlation between TFBUT and other tests: Osm (*r^2^* = 0.00), ST (*r^2^* = 0.06), fluorescein corneal staining (*r^2^* = 0.08), lissamine green conjunctival staining (*r^2^* = 0.21), and OSDI (*r^2^* = 0.05). In another relevant study by Inomata et al. [53], TFBUT was correlated to maximum blink interval (MBI) (*r* = 0.464), ST (*r* = 0.188), dry eye-related quality-of-life score (*r* = −0.106), and corneal fluorescein staining (*r* = −0.298). These studies revealed *r^2^* (or *r*) > 0.2 for OSS using fluorescein and/or lissamine green, like what we observed in our study. The use of MBI [54] and video capture manual analysis (VCMA) method-measured BI [55] were also shown to be better associated with TFBUT than BI.

There was a weak correlation between OSDI and clinical tests. OSDI is one of the most widely used survey instruments worldwide for recording patient symptoms [56], whereas clinical tests are applied for evaluating signs [57]. The measurement of patient symptoms and signs is a critical aspect in the evaluation of DED. Although this symptom-based questionnaire has been applied for screening, diagnostic, and evaluation purposes [58,59,60,61,62], the clinical signs in DED patients may be more clinically relevant [53]. Therefore, in line with our findings, either weak or no association between the OSDI score and results from the commonly used clinical DED tests has been previously reported [52,63,64,65].

A possible explanation for weak correlations in our study may be the lack of adequate reproducibility and accuracy of TFBUT [65,66], as well as other tests [5,66]. The order of tests performed, the skill level of practitioners, cooperation of patients, and properties and volume of fluorescein applied might have also influenced the TFBUT analyses [9,16]. A limitation of this study was that the stratification of data based on the same tests used for analyses might have caused bias. However, the diagnosis of DED needs a broader clinical panel than can be achieved with a single test. Because not all clinicians have access to a broad panel of tests or have time to perform, analyses of the predictive value of a single test measure (e.g., TFBUT) is of particular value.

On the question of sex difference (Table 5), only OSDI, Osm, and OSS grouped both below and above TFBUT cut-off of 2 s could detect significantly different values (*p* < 0.05). For Osm, BI, and ME, only groups below four cut-offs of TFBUT (≤2 s to ≤15 s) significantly determined the sex difference, as compared to OPI (≤5 s to ≤15 s). Our recent study [23] using three ST cut-off values (≤5 mm, ≤10 mm, and ≤15 mm) for grouping other ophthalmic tests was unable to detect any clear pattern of sex differences. Similar to the present study, the female sex has been linked to less stable tear film as well as an increased severity and frequency of dryness symptoms in comparison with male sex [44]. Several factors, including low androgen and high estrogen levels in reduced lacrimal and meibomian gland function, have previously been pointed out in addition to the use of eye makeup products by females [67,68,69,70,71,72,73,74].

## 7. Conclusions

The current study is shedding new light on tear film dynamics and demonstrates that TFBUT, to some extent. can discriminate pathological scores for some clinical dry eye tests, except the OSDI questionnaire and osmolarity. The ocular protection index is of particular interest, as it predicts the risk of corneal exposure and potential accompanying ocular surface inflammation. The authors support the Dry Eye Workshop II guidelines with a cut-off value of 10 s, but recommend a more stringent cut-off value of 2.5 s in order to robustly discriminate OPI. Further studies are warranted in order to explore the optimal cut-off value of TFBUT and discriminative power of other related dry eye tests. Additionally, investigating a set of clinical tests rather than one single examination in DED diagnosis is also needed for future research.

## Figures and Tables

**Table 1:** Test characteristics: overview of sample size for each test, out of 2094 recruited patients.

	Number of Subjects				
Test ^*^	Total	Female	Male	Missing Sex	Adjusted *p*-Value ^**^
OSDI	1977	1436	538	3	**0.00192**
Osm	835	633	201	1	**<0.001**
TFBUT	1959	1423	533	3	**<0.001**
BI	1971	1433	535	3	**0.00827**
OPI	1971	1433	535	3	0.214
ST	1942	1408	532	2	0.990
OSS	1959	1424	532	3	<0.001
ME	1873	1348	523	2	1.927
MQ	1871	1343	526	2	1.752
MGD	1881	1353	526	2	5.925

* Abbreviations: Ocular surface disease index (OSDI), osmolarity (Osm), tear film break-up time (TFBUT), blink interval (BI), ocular protection index (OPI), Schirmer I test (ST), ocular surface staining (OSS), meibum expressibility (ME), meibum quality (MQ), and meibomian gland dysfunction (MGD). ** *p*-value refers to differences in the test distribution within sex groups (Mann-Whitney test). *p*-values are adjusted for multiple testing according to the Bonferroni method. Bold values denote statistical significance at the *p* < 0.05 level.

**Table 2:** Subject demographics: age distribution, also sex stratified, with test for age group proportion being different in the two sex groups (Chi-square test).

	Number of Subjects			
Age Group	Total	Female	Male	Adjusted *p*-Value ^*^
0–19	28	11	17	**<0.001**
20–39	451	272	179	**<0.001**
40–59	783	599	184	**0.0107**
60–79	679	520	159	**0.0258**
80–99	78	64	14	0.3775
Total	2061 ^**^	1496	565	

^*^p-value refers to differences in the age group proportion within sex groups (Chi-squared test). *p*-values were adjusted for multiple testing according to the Bonferroni method. Bold values denote statistical significance at the *p* < 0.05 level. ^**^ Out of 2094 patients, 2019 have measurements of both age and sex, 42 of sex only, 3 of age only and 30 of neither age nor sex.

**Table 3:** Distribution of tear film break-up time (TFBUT) above and below each cut-off value.

	Cut-Off Value: 2		Cut-Off Value: 5		Cut-Off Value: 10		Cut-Off Value: 15	
	TFBUT≤2	TFBUT≥3	TFBUT≤5	TFBUT≥6	TFBUT≤10	TFBUT≥11	TFBUT≤15	TFBUT≥16
Mean±SD	1.56±0.501	6.06±3.912	2.56±1.279	9.64±4.156	3.44±2.310	15.4±3.713	3.81±2.940	19.73±2.388
*n*	824	1111	1502	433	1820	115	1894	41
Percent	42.58%	57.42%	77.62%	22.38%	94.06%	5.94%	97.88%	2.12%

**Table 4:** Comparison of clinical parameter results between groups based on the four defined cut-off values (2, 5, 10, and 15) for pathological dry eye disease (DED) measured by TFBUT (mean ± standard deviation, range and sample size; *p*-value of Mann–Whitney test of comparison for continuous variables and Chi-Square test for dichotomous one—meibomian gland dysfunction (MGD)).

	Cut-Off Value: 2			Cut-Off Value: 5			Cut-Off Value: 10			Cut-Off Value: 15		
Test *	TFBUT≤2	TFBUT≥3	p **	TFBUT≤5	TFBUT≥6	p **	TFBUT≤10	TFBUT≥11	p **	TFBUT≤15	TFBUT≥16	p **
OSDI	35.15±22.333 (0–100) 778	31.99±21.828 (0–100) 1004	**<0.001**	34.13±22.008 (0–100) 1392	30.64±22.239 (0–100) 390	**0.00336**	33.95±22.129 (0–100) 1678	23.98±19.431 (0–82) 104	**<0.001**	33.50±22.1 (0–100) 1748	26.6±21.285 (0–78.6) 34	0.0583
Osm	314.6±20.958 (276–395) 364	310.6±18.053 (275–398) 469	**0.0123**	312.9±19.837 (275–398) 663	310.3±17.852 (280–386) 170	0.124	312.5±19.466 (275–398) 798	307.9±19.203 (280–369) 35	0.139	312.38±19.49 (275–398) 820	310.46±18.496 (283–358) 13	0.737
BI	2.93±3.557 (0.8–60) 753	3.72±4.696 (1–60) 921	**<0.001**	3.15±3.924 (0.8–60) 1311	4.15±5.151 (1–60) 363	**<0.001**	3.33±4.276 (0.8–60) 1580	3.89±3.545 (1–30) 94	**<0.001**	3.35±4.266 (0.8–60) 1641	4.00±2.541 (1–15) 33	**<0.001**
OPI	0.701±0.386 (0–2) 753	2.299±1.825 (0.05–20) 921	**<0.001**	1.065±0.744 (0–5) 1311	3.441±2.291 (0.12–20) 363	**<0.001**	1.36±1.175 (0–10) 1580	5.25±2.767 (0.4–20) 94	**<0.001**	1.48±1.377 (0–11.82) 1641	6.33±3.374 (1.33–20) 33	**<0.001**
ST	11.65±8.755 (0–36) 799	16.14±10.173 (0–36) 1057	**<0.001**	13.20±9.417 (0–36) 1453	17.85±10.467 (0–36) 403	**<0.001**	13.99±9.729 (0–36) 1750	17.83±10.960 (1–36) 106	**<0.001**	14.1±9.791 (0–36) 1820	19.61±10.911 (4–36) 36	**0.00178**
OSS	2.48±2.418 (0–13) 823	1.50±1.822 (0–10) 1109	**<0.001**	2.15±2.239 (0–13) 1500	1.12±1.576 (0–9) 432	**<0.001**	1.99±2.176 (0–13) 1817	0.73±1.202 (0–6) 115	**<0.001**	1.95±2.161 (0–13) 1891	0.39±0.586 (0–2) 41	**<0.001**
ME	1.90±0.810 (0–3) 787	1.60±0.948 (0–3) 1051	**<0.001**	1.81±0.866 (0–3) 1428	1.45±0.976 (0–3) 410	**<0.001**	1.76±0.889 (0–3) 1728	1.3±1.019 (0–3) 110	**<0.001**	1.74±0.897 (0–3) 1798	1.3±1.091 (0–3) 40	**0.00733**
MQ	9.10±5.032 (0–24) 779	8.22±4.990 (0–24) 1046	**<0.001**	8.83±5.097 (0–24) 1418	7.80±4.687 (0–24) 407	**0.0022**	8.72±5.038 (0–24) 1717	6.63±4.402 (0–18) 108	**<0.001**	8.64±5.039 (0–24) 1785	6.62±3.953 (0–16) 40	**0.01795**
MGD	(0–1) 794	(0–1) 1062	**<0.001**	(0–1) 1441	(0–1) 415	**<0.001**	(0–1) 1744	(0–1) 112	**<0.001**	(0–1) 1816	(0–1) 40	0.06897

* Abbreviations: Ocular surface disease index (OSDI), osmolarity (Osm), tear film break-up time (TFBUT), blink interval (BI), ocular protection index (OPI), Schirmer I test (ST), ocular surface staining (OSS), meibum expressibility (ME), meibum quality (MQ) and meibomian gland dysfunction (MGD). ** *p*-value refers to differences in the test distribution between groups based on the corresponding TFBUT cut-off value. Bold values denote statistical significance at the *p* < 0.05 level. MGD is dichotomous (0 and 1), and therefore mean ± standard deviation is not defined in this case.

**Table 5:** Sex difference for each clinical parameter, and in each group below and above the four defined cut-off values (2, 5, 10, and 15) for pathological DED measured by TFBUT (mean ± standard deviation, Mann–Whitney test of comparison for continuous variables and Chi-Square test for dichotomous one—MGD).

		Cut-Off Value: 2		Cut-Off Value: 5		Cut-Off Value: 10		Cut-Off Value: 15	
Test ^*^		TFBUT≤2	TFBUT≥3	TFBUT≤5	TFBUT≥6	TFBUT≤10	TFBUT≥11	TFBUT≤15	TFBUT≥16
OSDI	F M *p* *	36.4±22.5 (0.100) 30.6±21.2 (0–97.9) **0.00197**	33.1±22.6 (0–100) 29.5±19.9 (0–83.3) **0.0458**	35.4±22.3 (0–100) 30.0±20.4 (0–97.9) **0.00016**	31.2±23.4 (0–100) 29.8±20.5 (0–83.3) 0.81	35.2±22.6 (0–100) 30.5±20.2 (0–97.9) **0.0004**	23.1±17.8 (0–75) 25.0±21.3 (0–82) 0.79	34.7±22.6 (0–100) 30.1±20.3 (0–97.9) **0.0003**	26.8±21.3 (2.1–75) 26.4±21.9 (0–78.6) 0.86
Osm	F M *p* *	316±21 (276 –395) 309±19 (281 –380) **0.00137**	312±19 (275–398) 307±15 (280–366) **0.0233**	314±20.2 (275– 398) 309±18.0 (281– 380) **0.0007**	312±19.4 (282– 386) 307±14.4 (280– 358) 0.10	314±20.1 (275– 398) 308±16.8 (281– 380) **0.0002**	311±19.6 (284–369) 305±18.8 (280–358) **0.23**	314±20.1 (275–398) 308±16.7 (280–380) **0.00005**	307±12.5 (289–324) 313±22 (283–358) 0.83
BI	F M *p* *	2.91±3.78 (0.8–60) 3.01±2.64 (1–30) 0.0544	3.49±3.61 (1–60) 4.21±6.53 (1–60) **0.046**	3.01±3.41 (0.8–60) 3.60±5.25 (1–60) **0.0008**	4.15±4.70 (1–60) 4.11±5.79 (1–60) 0.38	3.19±3.74 (0.8–60) 3.73±5.52 (1–60) **0.0022**	3.82±2.77 (1–15) 3.99±4.42 (1.1–30) 0.87	3.19±3.71 (0.8–60) 3.77±5.51 (1–60) **0.0016**	4.51±3.26 (1–15) 3.39±1.05 (2.1–6) 0.32
OPI	F M *p* *	0.719±0.403 (0–2) 0.637±0.311(0.07–2) 0.0699	2.19±1.7 (0.12–20) 2.56±2.06 (.05–12) **0.0465**	1.08±0.75 (0–5) 1.00±0.71 (.05–4.2) 0.0577	3.32±2.27 (0.12– 20) 3.65±2.32 (0.15– 12) 0.198	1.32±1.09 (0–9.1) 1.49±1.40 (.05–10) 0.34	5.18±2.96 (0.9–20) 5.34±2.52 (0.4–12) 0.51	1.42±1.25 (0–10.8) 1.67±1.66 (.05–11.8) 0.11	6.14±4.32 (1.3– 20) 6.57±1.81 (3.3– 9.5) 0.14
ST	F M *p* *	11.8±8.87 (0–36) 11.1±8.35 (0–36) 0.55	15.9±10.4 (0–36) 16.6±9.72 (0–36) 0.13	13.3±9.58 (0–36) 13.0±8.87 (0–36) 0.81	17.7±10.7 (0–36) 18.1±10.2 (1–36) 0.54	13.9±9.85 (0–36) 14.2±9.41 (0–36) 0.25	17.1±11.3 (1–36) 18.7±10.6 (1–36) 0.32	14.0±9.87 (0–36) 14.5±9.59 (0–36) 0.12	20.1±12.3 (4–36) 19.1±9.31 (7–35) 0.91
OSS	F M *p* *	2.57±2.47 (0–12) 2.15±2.22 (0–13) **0.0401**	1.58±1.85 (0–10) 1.32±1.74 (0–10) **0.0072**	2.22±2.29 (0–12) 1.92±2.06 (0–13) **0.0372**	1.20±1.57 (0–7) 0.99±1.57 (0–9) 0.0593	2.08±2.23 (0–12) 1.73±2.00 (0–13) **0.0017**	0.87±1.27 (0–6) 0.55±1.10 (0–6) **0.0828**	2.05±2.22 (0–12) 1.67±1.98 (0–13) **0.0003**	0.52±0.60 (0–2) 0.25±0.55 (0–2) 0.0869
ME	F M *p* *	1.89±0.792 (0–3) 1.93±0.871 (0–3) 0.39	1.63±0.936 (0–3) 1.54±0.971 (0–3) 0.15	1.79±0.854 (0–3) 1.85±0.902 (0–3) 0.20	1.55±0.977 (0–3) 1.31±0.958 (0–3) **0.0162**	1.77±0.869 (0–3) 1.72±0.941 (0–3) 0.48	1.34±1.06 (0–3) 1.25±0.98 (0–3) 0.72	1.76±0.876 (0–3) 1.69±0.949 (0–3) 0.27	1.3±1.17 (0–3) 1.3±1.03 (0–3) 0.99
MQ	F M *p* *	8.96±4.95 (0–24) 9.57±5.26 (0–24) 0.15	8.11±4.97 (0–24) 8.42±4.99 (0–24) 0.29	8.69±5.08 (0–24) 9.23±5.14 (0–24) 0.0714	7.61±4.42 (0–24) 8.01±4.97 (0–22) 0.59	8.59±4.99 (0–24) 9.06±5.11 (0–24) 0.0693	6.51±4.11 (0–16) 6.76±4.75 (0–18) 0.94	8.53±4.99 (0–24) 8.91±5.14 (0–24) 0.13	6.4±3.87 (0–12) 6.85±4.12 (0–16) 0.95
MGD	F M *p* *	(0–1) (0–1) 1	(0–1) (0–1) 1	(0–1) (0–1) 0.19	(0–1) (0–1) 0.40	(0–1) (0–1) 0.84	(0–1) (0–1) 0.77	(0–1) (0–1) 0.6	(0–1) (0–1) 1

* Abbreviations: Ocular surface disease index (OSDI), osmolarity (Osm), tear film break-up time (TFBUT), blink interval (BI), ocular protection index (OPI), Schirmer I test (ST), ocular surface staining (OSS), meibum expressibility (ME), meibum quality (MQ), and meibomian gland dysfunction (MGD). *p*-value refers to differences in the test distribution within sex groups. Bold values denote statistical significance at the *p* < 0.05 level. MGD is dichotomous (0 and 1) and, therefore, mean ± standard deviation is not defined in this case.

**Table 6:** Pearson’s correlation coefficient of determination (*r*) of TFBUT to all other clinical test.

Variable *	Pearson’s *r* to TFBUT	95% CI for *r*	*p*-Value **
OSDI	−0.091	(−0.143, −0.051)	**<0.0001**
Osm	−0.049	(−0.116, 0.011)	0.0527
BI	0.093	(0.053, 0.148)	**<0.0001**
OPI	0.696	(0.677, 0.718)	**<0.0001**
ST	0.205	(0.162, 0.249)	**<0.0001**
OSS	−0.241	(−0.283, −0.199)	**<0.0001**
ME	−0.191	(−0.238, −0.150)	**<0.0001**
MQ	−0.108	(−0.155, −0.065)	**<0.0001**
MGD	−0.134	(−0.180, −0.091)	**<0.0001**

* Abbreviations: Ocular surface disease index (OSDI), osmolarity (Osm), tear film break-up time (TFBUT), blink interval (BI), ocular protection index (OPI), Schirmer I test (ST), ocular surface staining (OSS), meibum expressibility (ME), meibum quality (MQ) and meibomian gland dysfunction (MGD). ** *p*-value refers to the test on the Pearsons’ correlation coefficient being different from 0. Bold values denote statistical significance at the *p* < 0.05 level.

**Table 7:** Pearson’s correlation coefficient of determination (*r*).

	OSDI	Osm	BI	OPI	ST	OSS	ME	MQ	MGD
OSDI	1	0.036	−0.042	−0.004	−0.057	0.09	0.009	−0.002	0.033
Osm	0.036	1	−0.066	−0.003	−0.061	0.082	−0.045	−0.035	−0.031
BI	−0.042	−0.066	1	−0.189	0.015	−0.079	−0.11	−0.028	−0.038
OPI	−0.004	−0.003	−0.189	1	0.138	−0.152	−0.176	−0.075	−0.121
ST	−0.057	−0.061	0.015	0.138	1	−0.193	−0.004	−0.015	0.011
OSS	0.09	0.082	−0.079	−0.152	−0.193	1	0.107	−0.004	0.058
ME	0.009	−0.045	−0.11	−0.176	−0.004	0.107	1	0.173	0.289
MQ	−0.002	−0.035	−0.028	−0.075	−0.015	−0.004	0.173	1	0.259
MGD	0.033	−0.031	−0.038	−0.121	0.011	0.058	0.289	0.259	1

Abbreviations: Ocular surface disease index (OSDI), osmolarity (Osm), tear film break-up time (TFBUT), blink interval (BI), ocular protection index (OPI), Schirmer I test (ST), ocular surface staining (OSS), meibum expressibility (ME), meibum quality (MQ) and meibomian gland dysfunction (MGD).

## Data Availability

Data sharing not applicable.
